# Supplementary material for: Role and mechanism of NCAPD3 in promoting malignant behaviors in gastric cancer
Source: Front Pharmacol. 2024 Apr 22;15:1341039. doi: 10.3389/fphar.2024.1341039 (PMC11070777; doi:10.3389/fphar.2024.1341039)
Supplement: Supplementary file 11 [file DataSheet2.ZIP › GSEA/GO_CC/my_analysis.Gsea.1599461408869/GO_EXTRACELLULAR_MATRIX.html]

Details for gene set GO\_EXTRACELLULAR\_MATRIX[GSEA]

|  || Dataset | filtered\_dataset.sample\_info.cls#WT\_versus\_NCAPD3\_MUT |
| Phenotype | sample\_info.cls#WT\_versus\_NCAPD3\_MUT |
| Upregulated in class | NCAPD3\_MUT |
| GeneSet | GO\_EXTRACELLULAR\_MATRIX |
| Enrichment Score (ES) | -0.36313072 |
| Normalized Enrichment Score (NES) | -1.989617 |
| Nominal p-value | 0.0024937657 |
| FDR q-value | 0.04287573 |
| FWER p-Value | 0.115 |
Table: GSEA Results Summary

  

Fig 1: Enrichment plot: GO\_EXTRACELLULAR\_MATRIX      
 Profile of the Running ES Score & Positions of GeneSet Members on the Rank Ordered List

  

| SYMBOL | TITLE | RANK IN GENE LIST | RANK METRIC SCORE | RUNNING ES | CORE ENRICHMENT || 1 | 7052 | TGM2 | 17 | 1.045 | 0.0392 | No |
| 2 | 2243 | FGA | 18 | 1.039 | 0.0904 | No |
| 3 | 4811 | NID1 | 51 | 0.913 | 0.1121 | No |
| 4 | 64856 | VWA1 | 259 | 0.623 | -0.0075 | No |
| 5 | 2244 | FGB | 321 | 0.582 | -0.0231 | No |
| 6 | 8573 | CASK | 368 | 0.554 | -0.0292 | No |
| 7 | 1755 | DMBT1 | 480 | 0.487 | -0.0858 | No |
| 8 | 23767 | FLRT3 | 781 | 0.333 | -0.2872 | No |
| 9 | 4670 | HNRNPM | 790 | 0.330 | -0.2768 | No |
| 10 | 1605 | DAG1 | 823 | 0.304 | -0.2850 | No |
| 11 | 667 | DST | 886 | -0.303 | -0.3151 | No |
| 12 | 301 | ANXA1 | 914 | -0.342 | -0.3179 | No |
| 13 | 3913 | LAMB2 | 919 | -0.348 | -0.3036 | No |
| 14 | 4052 | LTBP1 | 928 | -0.353 | -0.2920 | No |
| 15 | 3914 | LAMB3 | 1015 | -0.414 | -0.3341 | No |
| 16 | 1191 | CLU | 1056 | -0.450 | -0.3409 | Yes |
| 17 | 6590 | SLPI | 1062 | -0.453 | -0.3223 | Yes |
| 18 | 1308 | COL17A1 | 1065 | -0.455 | -0.3013 | Yes |
| 19 | 283 | ANG | 1074 | -0.458 | -0.2845 | Yes |
| 20 | 3918 | LAMC2 | 1088 | -0.466 | -0.2710 | Yes |
| 21 | 5359 | PLSCR1 | 1146 | -0.511 | -0.2872 | Yes |
| 22 | 1282 | COL4A1 | 1165 | -0.534 | -0.2740 | Yes |
| 23 | 2152 | F3 | 1188 | -0.559 | -0.2624 | Yes |
| 24 | 145864 | HAPLN3 | 1238 | -0.616 | -0.2676 | Yes |
| 25 | 2734 | GLG1 | 1240 | -0.618 | -0.2379 | Yes |
| 26 | 7057 | THBS1 | 1242 | -0.620 | -0.2080 | Yes |
| 27 | 1893 | ECM1 | 1250 | -0.637 | -0.1817 | Yes |
| 28 | 4240 | MFGE8 | 1280 | -0.668 | -0.1698 | Yes |
| 29 | 5046 | PCSK6 | 1297 | -0.699 | -0.1470 | Yes |
| 30 | 84947 | SERAC1 | 1303 | -0.707 | -0.1158 | Yes |
| 31 | 1116 | CHI3L1 | 1339 | -0.777 | -0.1030 | Yes |
| 32 | 7422 | VEGFA | 1346 | -0.788 | -0.0685 | Yes |
| 33 | 59277 | NTN4 | 1390 | -0.999 | -0.0505 | Yes |
| 34 | 4312 | MMP1 | 1407 | -1.304 | 0.0022 | Yes |
Table: GSEA details [plain text format]

  

Fig 2: GO\_EXTRACELLULAR\_MATRIX      
 Blue-Pink O' Gram in the Space of the Analyzed GeneSet

  

Fig 3: GO\_EXTRACELLULAR\_MATRIX: Random ES distribution      
 Gene set null distribution of ES for **GO\_EXTRACELLULAR\_MATRIX**
